# Transfusion-Transmitted Hepatitis E: NAT Screening of Blood Donations and Infectious Dose

**DOI:** 10.3389/fmed.2018.00005

**Published:** 2018-02-01

**Authors:** Jens Dreier, Cornelius Knabbe, Tanja Vollmer

**Affiliations:** ^1^Institut für Laboratoriums- und Transfusionsmedizin, Herz- und Diabeteszentrum Nordrhein- Westfalen, Universitätsklinik der Ruhr-Universität Bochum, Bad Oeynhausen, Germany

**Keywords:** hepatitis E virus, blood donor, blood safety, NAT testing, transfusion–transmission

## Abstract

The risk and importance of transfusion-transmitted hepatitis E virus (TT-HEV) infections by contaminated blood products is currently a controversial discussed topic in transfusion medicine. The infectious dose, in particular, remains an unknown quantity. In the present study, we illuminate and review this aspect seen from the viewpoint of a blood donation service with more than 2 years of experience in routine HEV blood donor screening. We systematically review the actual status of presently known cases of TT-HEV infections and available routine NAT-screening assays. The review of the literature revealed a significant variation regarding the infectious dose causing hepatitis E. We also present the outcome of six cases confronted with HEV-contaminated blood products, identified by routine HEV RNA screening of minipools using the highly sensitive RealStar HEV RT-PCR Kit (95% LOD: 4.7 IU/mL). Finally, the distribution of viral RNA in different blood components [plasma, red blood cell concentrate (RBC), platelet concentrates (PC)] was quantified using the first WHO international standard for HEV RNA for NAT-based assays. None of the six patients receiving an HEV-contaminated blood product from five different donors (donor 1: RBC, donor 2–5: APC) developed an acute hepatitis E infection, most likely due to low viral load in donor plasma (<100 IU/mL). Of note, the distribution of viral RNA in blood components depends on the plasma content of the component; nonetheless, HEV RNA could be detected in RBCs even when low viral plasma loads of 100–1,000 IU/mL are present. Comprehensive retrospective studies of TT-HEV infection offered further insights into the infectivity of HEV RNA-positive blood products. Minipool HEV NAT screening (96 samples) of blood donations should be adequate as a routine screening assay to identify high viremic donors and will cover at least a large part of viremic phases.

## Introduction

Hepatitis E virus (HEV) is an emerging infectious threat to blood safety. In the recent decade, there have been several reports of transfusion-transmitted hepatitis E virus (TT-HEV) infection [for review, see Ref. (1)], although the risk of infection through consumption of raw or undercooked pork and wild boar is even greater (2). In industrialized countries, HEV infection, mainly with genotype 3, usually causes an acute, self-limiting, asymptomatic, or mild hepatitis. However, the significance of HEV genotype 3 infections has changed because chronic hepatitis with rapidly progressive cirrhosis in organ transplant recipients and patients with hematological malignancy, as well as fulminant hepatitis in patients with chronic liver disease, have been observed (1, 3). HEV-infected immunocompromised patients develop chronic hepatitis E in approximately 60% of cases (4).

Since 2004, HEV has gained importance as a transfusion transmissible infectious agent, although earlier reports pointed to the risk of infection (5, 6). HEV has been transmitted in samples of red blood cells, platelet preparations, pooled granulocytes, and fresh frozen plasma (FFP) (solvent-detergent treated, amotosalen-treated, secured by quarantine). As a result, the public health implications of HEV in Europe have gained greater momentum due to an increasing number of hepatitis E cases and recent reports of chronic, persistent HEV infections associated with progression to cirrhosis in immunosuppressed patients. The question of hepatitis E and contribution of NAT screening on blood safety is currently extensively discussed, not only by several European committees and local blood authorities but also internally by a large number of blood transfusion facilities. Domanovic questioned the situation as “a shift to screening” and summarized the epidemiology of HEV infections among blood donors and outlined strategies to prevent TT-HEV in 11 European countries (7). A nationwide HEV RNA screening of blood donations was introduced in Ireland, the UK, and recently the Netherlands. Several blood establishments in Germany, France, and recently Switzerland perform selective screening intended for use in high-risk patients or universal screening for HEV RNA. Blood authorities in Greece, Portugal, Italy, and Spain are evaluating the situation (7). Regardless of the risk of HEV transmission *via* blood products, most authorities have recommended HEV monitoring of immunosuppressed patients. The implementation of a HEV run control for screening human plasma pools requested by the Ph. Eur. 1646 (8) is another indication toward a transfusion relevance of HEV. So far, there have been only specific case reports of HEV transmission by SD-treated plasma (SDP) but not by other plasma-derived medicinal products. However, it is not unlikely that cases might have been overlooked due to diagnostic failure (9). Past serologic investigations in Japan implicated coagulation factors in the transmission of HEV. The conclusion was based on the significantly higher prevalence of HEV antibody in hemophilia patients receiving coagulation factors that were not subjected to virus inactivation or removal, compared with patients who received virus-inactivated coagulation factors (10). Cost-effectiveness analyses were carried out in the Netherlands to assess whether an appropriate measure should be implemented for blood donor screening (11). The analysis led to the conclusion that the prevention of HEV transmission through the screening of blood donations is not markedly expensive compared with other blood-screening measures. However, the key issue of these cost-effectiveness analyses is the minimum viral load required to be detected in the donor blood. Thus, attention is now focused on the limit of detection of NAT (ID versus pool NAT), which is primary influenced by the minimum infectious dose of a blood product triggering an infection in the recipient. The German Advisory Committee on Blood (Arbeitskreis Blut) recommended a NAT sensitivity of 100 IU HEV RNA/mL [per single donation (12)], which is difficult to achieve with the currently available NAT assays using minipool NAT. For these reasons, the ongoing discussions address the question of the most appropriate and effective strategy to minimize the risk of TT-HEV infection, taking into account the costs, the logistics of testing, and the infection risk and outcome of HEV-infected blood recipients. The present review provides a comprehensive view of the various aspects of TT-HEV infection and a discussion on the current status on the issue of screening for this virus.

## Materials and Methods

### HEV RNA Screening, Serological Testing, and Measurement of Liver-Specific Parameters in Blood Donors and Transfusion Recipients

Routine HEV RNA screening of therapeutic blood products was introduced in our blood donation service in January 2015. From January 2015 to July 2017, a total of 235,524 donations from 86,933 donors were screened for the presence of HEV RNA revealing 182 HEV RNA-positive donors. For four of these HEV RNA-positive donors, a lookback procedure need to be initiated, and a total of nine viremic previous donations of these donors were identified, which were transfused to six different recipients (Table 1).

**Table 1:** Cases of transfusion of blood products containing hepatitis E virus (HEV) RNA from this study.

Donor					Recipient					Outcome			
Donor	Blood product	Viral load (IU/mL), genotype	Infectious dose (IU)	Anti-HEV IgM/IgG	Recipient, sex and age		Anti-HEV IgG^a^	Primary disease	Immuno-compromised	Follow-up period (days)	Outcome	HEV-PCR	Anti-HEV IgG
1	RBC (314 mL)	<25 GT 3	<2.50E+02	Negative	1	M, 23 years	Negative	Heart transplantation	Yes	134 days	No HEV infection	Negative	Negative

2	PC 1 (234 mL)	<25 GT 3	<4.68E+03	Negative	2	M, 76 years	Negative	Heart valve failure, atrial fibrillation	No	35 days	No HEV infection, died sepsis	Negative	Negative
	PC 2 (243 mL)		<4.86E+03		3	M, 54 years		Left ventricular heart failure	No	50 days	No HEV infection	Negative	Negative

3 donation 1	PC 1 (230 mL)	27.8 GT 3	5.12E+03	Negative	4	F, 26 years	Negative	Hypertrophic cardiomyopathy	No	16 days	No HEV infection^b^	Negative	Negative
	PC 2 (254 mL)		5.65E+03										
	Total		1.08E+04										

3 donation 2	PC 1 (244 mL)	69.4 GT 3	1.35E+04	Negative	5	M, 72 years	Negative	Arrhythmia	No	NA	Died arrhythmia	NA	NA
	PC 2 (244 mL)		1.35E+04										
	Total		2.71E+04										

4	PC 1 (242 mL)	<25 GT 3	<3.97E+03	Negative	6	F, 79 years	Negative	Leukemia	Yes	NA	Died leukemia	NA	NA
	PC 2 (244 mL)		<4.00E+03										
	Total		7.97E+03										

*^a^At the date of transfusion*.

*^b^Patient no longer available*.

Hepatitis E virus RNA-positive blood donors were identified using the RealStar HEV RT-PCR Kit (Altona Diagnostics, Hamburg, Germany), as described previously (13). Total nucleic acid (RNA/DNA) was extracted from 500 µL of donor and recipient samples using the NucliSens easyMAG (bioMerieux, Nürtingen, Germany) automated RNA/DNA extraction system followed by HEV RNA detection using the RealStar HEV RT-PCR Kit (13). HEV titer of positive samples was quantified using the first WHO international standard for HEV RNA for NAT-based assays (Paul Ehrlich Institute, Langen, Germany). The linear range of quantification was from 25 to 10E+07 IU/mL.

Screening for the presence of HEV-specific IgM and IgG antibodies was performed using the Anti-HEV ELISA (IgM and IgG, Euroimmun, Lübeck, Germany) according to the manufacturer’s instructions. Serum concentrations of glutamate dehydrogenase (GLDH), aspartate aminotransferase (AST), alanine aminotransferase (ALT), and total bilirubin were measured in plasma samples using the respective enzymatic assays on the Architect system (Abbott Diagnostics Europe, Wiesbaden, Germany). All HEV-infected donors underwent pre-donation medical examination and negated current diseases or any known risk factors for viral infection. The study protocol followed the ethical guidelines of the Ruhr University, Bochum, and was approved by the institutional review board; donors provided informed consent.

### Processing of Blood Products and Quantification of the Viral Load

Apheresis-derived single-donor PCs (APCs) were prepared after standard processing with the Haemonetics MCS + (Haemonetics GmbH, München, Germany). The final product consisted of 2.0–4.0 × 10E+11 platelets/unit (205–295 mL) containing 0.76–0.84 mL/mL human plasma and 0.16–0.24 mL/mL ACD-A stabilizer. For the preparation of RBC, whole blood donations were collected into a multiple bag system with inline filtration for leukoreduction (CompoFlow quadruple 4F, 70-mL CPD/100-mL PAGGS-M—WB + PDS-V, Fresenius Kabi Deutschland GmbH, Bad Homburg, Germany), followed by centrifugation of the filtered whole blood unit at 4,182 × *g* and 22°C for 45 min. Automated fractionation was carried out using the CompoMat G5 separator (Fresenius Kabi), and RBCs were stored directly at 4 ± 2°C. The final product volume averaged from 200 to 400 mL. The residual plasma volume was estimated to be 10 mL per RBC. The corresponding plasma products (FFP) contained a total volume of 180–380 mL with 0.75–0.82 mL/mL human plasma.

Viral RNA in the different blood components (FFP, RBC, RBC supernatant, PC) was extracted from 4.8-mL sample with the Chemagic Viral DNA/RNA reagent kit (Viral 5k, PerkinElmer Chemagen Technology GmbH, Baesweiler, Germany) combined with the automated Chemagic magnetic separation module MSMI (PerkinElmer Chemagen Technology GmbH) according to the manufacturer’s instructions. For the recovery of RBC supernatant, 50 mL of RBC were transferred to EDTA-containing monovettes followed by centrifugation for 10 min at 5,000 rpm. Therefore, the RBC supernatant contained CPD/PAGGS-M stabilizator and a minimal proportion of residual plasma. For RNA extraction of RBCs, the alternative lysis buffer CMG-825 (lysis buffer blood, PerkinElmer Chemagen Technology GmbH) was used. The 95% lower limit of detection was calculated by Probit analysis to 4.7 IU/mL [confidence interval: 3.6–7.5 IU/mL (13)] for FFP, PC, and RBC supernatant and to 8.9 IU/mL (confidence interval: 6.5–21.1 IU/mL) for RBCs.

### Searching Criteria

For the systematic review of HEV cases, the PubMed database (http://www.ncbi.nlm.nih.gov/pubmed/), a public search engine maintained by the United States National Library of Medicine (NLM) at the National Institutes of Health (NIH) that provides access to over 24 million citations in all fields of life sciences, mostly from the MEDLINE (Medical Literature Analysis and retrieval System Online), was searched for publications between 2004 and 2017 (publications dates) using specific search strings including “hepatitis E/HEV infection,” “transfusion transmitted hepatitis E/HEV infection,” and “hepatitis E/HEV blood donor screening.”

### Statistical Analysis

All values are given as mean ± SD. Median values and SD were calculated and Spearman’s rank correlation analysis was performed using the GraphPad Prism 5.0 software (GraphPad Software, San Diego, CA, USA). Statistical analysis to assess differences between values was performed using the Mann–Whitney *U* test.

## Results

A systematic review of individual case reports regarding the transfusion of HEV-contaminated blood products from 2004 to 2017 is summarized in chronological order of occurrence in Table S1 in Supplementary Material. Cases including those patients who died shortly after transfusion for reasons other than HEV infection were excluded. We further describe six new cases of patients transfused with HEV-contaminated blood products, and none of the recipients developed HEV infection.

### Case Description

Table 1 summarizes the donor and recipient information of all six cases. Anti-HEV IgM and IgG antibodies were not detected in any HEV RNA-positive donor and serum concentrations of GLDH, AST, ALT, and total bilirubin were all within normal range (data not shown). The presence of HEV RNA was confirmed in a secondary sample. Additionally, the corresponding plasma product of the RBC of donor 1 was available and the presence of HEV RNA was further confirmed. Donors 1 and 4 did not return for blood donation after HEV infection. For the other two donors, anti-HEV IgG seroconversion was observed after 149 days (donor 2) and 116 days (donor 3) after the first HEV RNA-positive donation. For HEV genotyping of all donor samples, HEV-nucleotide sequence, corresponding to a 242-bp fragment of the ORF1 region, was amplified and sequenced. Phylogenetic analysis showed that the samples clustered together and were related to HEV genotype 3, which is prevalent in Germany.

All recipients were anti-HEV IgM and anti-HEV IgG negative at the time of transfusion of HEV RNA-positive blood products. The viral load in plasma samples of donors 1, 2, and 4 was determined to be 17, 12, and 20 IU/mL, respectively. These values were below the linear range of quantification (<25 IU/mL), and therefore a maximum infectious dose was calculated assuming a viral load of 25 IU/mL. Recipient 1, an immunocompromised man after heart transplantation, received one RBC. Assuming a residual plasma volume of 10 mL per RBC, the maximum corresponding infectious dose was calculated as 250 IU. This patient did not develop HEV infection within the follow-up period of 134 days, and neither HEV RNA nor anti-HEV antibodies were detectable.

Each apheresis platelet donation resulted in two APCs. The two immunocompetent recipients R2 and R3 received APCs from donor 2 with infectious doses of 4.68E+03 IU (APC1) and 4.86E+03 IU (APC2), assuming an average residual plasma volume of 0.8 mL per APC. Neither patients developed an HEV infection within the follow-up period of 35 days (recipient R2) or 50 days (recipient R3); moreover, no HEV RNA or anti-HEV antibodies were detectable. Accordingly, recipient R6 received two apheresis platelets (APC1: 242 mL, APC2: 244 mL) from donor 4 with a total maximum infectious dose of 7.97E+03 IU (total volume transfused: 486 mL), but she died shortly after transfusion for reasons other than HEV infection.

Donor 3 donated platelets regularly approximately every 14 days and showed the highest viral load compared with the other donors. The first viremic donation (donation 1) contained 27.8 IU/mL HEV RNA, and the second donation, 18 days later (donation 2), contained 69.4 IU/mL HEV RNA. The double APCs were transfused to two immunocompetent recipients (recipients R4 and R5). Recipient 4 received a total infectious dose of 1.08E+04 IU HEV RNA (donation 1, total volume of 484 mL, APC1: 230 mL, APC2: 254 mL). This patient did not develop an acute HEV infection within 16 days after transfusion, and neither HEV RNA nor anti-HEV antibodies were detectable. However, the observation period was short and the possibility that HEV infection may have occurred later could not be ruled out. This patient was released from hospital and unfortunately no follow-up samples were sent to our laboratory for further follow-up. The second HEV-positive donation of donor 3 had a total infectious dose corresponding to 2.71E+04 IU HEV RNA (donation 2, transfusion of a total volume of 488-mL APC, APC1, and APC2: 244 mL). The recipient (R5) of these two apheresis platelets died shortly after transfusion for reasons other than HEV infection.

The cases of recipients 4–6 were excluded from the subsequent overview due to the short follow-up period.

### Distribution of Viral RNA in Different Blood Products

**Figure 1:**
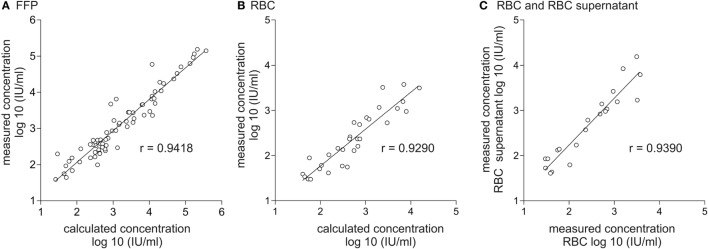
Correlation of calculated and effectively quantified viral load in fresh frozen plasma (FFP) and red blood cell concentrate (RBC) and correlation of viral load in RBC and RBC supernatant. Displayed is the correlation between the effectively quantified hepatitis E virus (HEV) titer and the expected viral load in FFP **(A)** and RBC **(B)**. Calculation of the expected viral load in FFP is based on quantification results of HEV viral load in plasma of donors assuming a mean plasma content of 0.8 mL/mL plasma in the corresponding plasma product. Calculation of the expected viral load in RBC is based on quantification results of HEV viral load in plasma of donors assuming a residual plasma content of 10 mL/RBC. **(C)** Correlation of the effectively quantified HEV titer in RBC and RBC supernatant. The linear range of quantification was from 25 to 10E+07 IU/mL. Therefore, all values <25 IU/mL were excluded.

In order to determine if a reduction of the viral load occurs during the manufacturing process of blood products, e.g., by centrifugation or by adsorption to components of the blood bag system, including the filter used for leukoreduction, viral loads were quantified in the plasma of HEV RNA-positive donors and additionally quantified in the corresponding blood products, FFP and RBC. Results from the respective blood products were correlated with the expected viral loads calculated with quantified results for plasma of HEV RNA-positive donors, assuming no removal during the production process. Calculation of virus titer assumed a mean plasma content of 0.80 mL/mL human plasma (80%) for FFP. For RBCs, the remaining plasma volume of 10 mL per RBC was assumed for consideration of the total volume of each individual RBC after processing. A total of 73 value pairs were available for correlation analysis of FFPs (Figure 1A), of which three were excluded due to low viral load (<25 IU/mL). Likewise, a total of 73 value pairs were available for RBC (Figure 1B), of which 31 with low viral load (<25 IU/mL) were excluded. Spearman’s correlation analysis revealed a good correlation of *r* = 0.9418 (95% CI: 0.9065–0.9641) for FFP and a good correlation of *r* = 0.9290 (95% CI: 0.8538–0.9663) for RBC. The wider distribution between the measured and calculated HEV titer in RBC is based on the considerably lower plasma amount of only 10 mL per RBC (mean RBC volume 268 mL, mean plasma proportion 3.7%) and the resultant higher method-specific quantification error.

In order to determine if HEV RNA or virus particles are bound to the surface of red blood cells, HEV RNA was quantified in 20 different RBCs as well as in the cell-free supernatants of RBCs (Figure 1C). Spearman’s correlation analysis again revealed a good correlation of *r* = 0.9390 (95% CI: 0.8492–0.9760), indicating that no specific binding to red blood cell surfaces had occurred.

**Figure 2:**
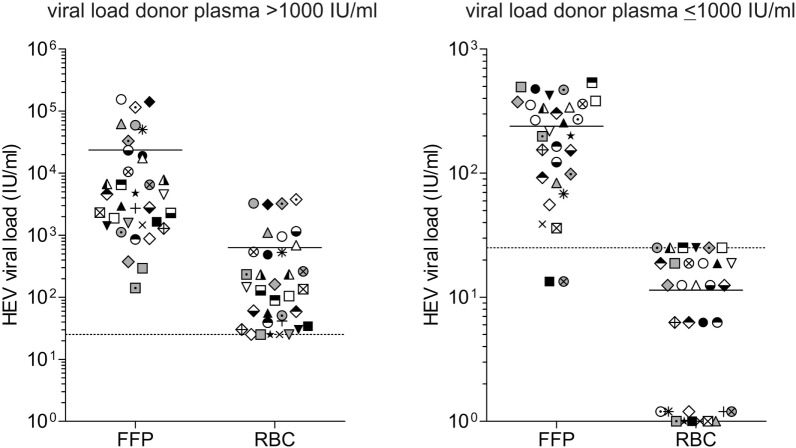
Distribution of viral load in different blood products. The hepatitis E virus (HEV) titer in plasma of donors and the corresponding blood products fresh frozen plasma (FFP) and red blood cell concentrates (RBCs) was quantified using the first WHO international standard for HEV RNA for NAT-based assays. The distinction of viral loads >1,000 IU/mL **(A)** and ≤1,000 IU/mL **(B)** is based on quantification results of HEV viral load in plasma of donors, not in the corresponding blood products. Equal symbols present quantification results in different blood products from the same donor, quantification was performed in quadruplicate. The linear range of quantification was from 25 to 10E+07 IU/mL. Values <25 IU/mL were displayed as 25 IU/mL. For RBCs with low viral loads, not all replicates were positive for HEV RNA. Results were displayed as follows: 0 IU/mL: 0/4 positive replicates; 6.25 IU/mL: 1/4 positive replicates; 12.5 IU/mL: 2/4 positive replicates; 18.75 IU/mL: 3/4 positive replicates; and 25 IU/mL: 4/4 positive replicates (quantification results <25 IU/mL). The solid horizontal line represents mean values, and the dotted horizontal line is representative for the value 25 IU/mL.

Figure 2 displays the distribution of viral RNA in FFP and RBC depending on the viral load, quantified in the plasma of HEV RNA-positive donors. HEV RNA was detected in the RBCs of all donations where the viral load in plasma was quantified as >1,000 IU/mL (Figure 2A). Quantified FFPs contained a total mean volume of 287 mL, corresponding to a total plasma volume of 230 mL. Comparison of the quantified mean values for FFP (2.34E+04 ± 4.08E+04 IU/mL) with those obtained for RBCs (6.29E+02 ± 1.05E+03 IU/mL) revealed the percentage proportion of 2.7% for RBCs, essentially corresponding to the calculated mean plasma proportion of 3.7%.

For RBCs, where the viral load in plasma was quantified >1,000 IU/mL (Figure 2B), a maximum viral load of 25 IU/mL was detected and often not all replicates were positive for HEV RNA. Negative results might either indicate that RBC contains no viral RNA or the viral load is below the detection limit of the assay (8.9 IU/mL).

## Discussion

German public health authorities have recognized an increasing number of acute HEV infections, which is probably due to higher clinical awareness but more likely due to detection of HEV-infected asymptomatic blood donors identified through NAT screening by blood donation establishments. A high frequency of HEV viremic donors have been reported in recent large screening studies in several European countries (13–17). The asymptomatic infection is mostly characterized by a period of asymptomatic viremia, with an estimated duration of 68 days (18). The typical serological course of an acute HEV infection showed detectable IgM antibodies following an incubation period of 2–6 weeks, decline to baseline levels within three to 6 month, followed by longer lasting IgG antibodies which remains detectable for up to 15 years (19–21). The progression of anti-HEV immunoglobulins in asymptomatic cases is comparable with symptomatic cases (22). However, the factual incidence of TT-HEV infection and the real clinical importance is currently unknown. The rate of reported TT-HEV infections is very small, probably due to underreporting, failure to recognize or misinterpretation of symptoms (23), or development of HEV infection long after transfusion, hampering any association with an earlier transfusion event (24). Moreover, the occurrence of primary asymptomatic infection in recipients is certainly an option. Besides a recent large study in England (24), only a small number of individual cases of TT-HEV infection (Table S1 in Supplementary Material) and cases of transfusion of HEV-containing blood without TT-HEV infection (Table S2 in Supplementary Material) have been described (14, 23, 25–46). The large 2012–2013 study in England retrospectively screened 225,000 English blood donors for HEV by NAT. Follow-up of the recipients who had received HEV genotype 3-contaminated blood components indicated that 42% had evidence of TT-HEV infection, with transmission possibly linked to the absence of HEV-specific antibodies (24). A high virus load in the donor, corresponding with the volume of plasma transfused with the final blood component, rendered infection more likely. Moreover, multiple different kinds of blood products were involved, but the transmission rates varied. Of all transfused RBC, only 25% caused HEV infection, whereas 40% of transfused PPCs, 50% of transfused APCs, and 100% of transfused FFPs or pooled granulocytes caused HEV infection (24).

Analysis of 19 Japanese cases of TT-HEV infection by Satake et al. found a comparable rate of infection of 50% (29). All TT-HEV cases present in the Satake’s study were included in our case analysis (Table S1 in Supplementary Material). The studies by Satake et al. and Hewitt et al. [18 patients (24)] also identified several patients transfused with HEV-contaminated components without the development of HEV infection [5 patients (29), 18 patients (24)], but these cases were not considered in Table S2 in Supplementary Material because no detailed case descriptions are available.

Unfortunately, some cases only revealed poor data sets, missing important facts for both, the recipients of contaminated blood products or the respective donors. For example, the pretransfusion serostatus in recipients is often only assumable based on the posttransfusion status, or the serostatus is entirely absent. The serological status of the blood donors is also often missing. Additionally, the duration between transfusion, determination of infection, and follow-up of patients including the accompanying therapy and laboratory parameters is often incomplete or untraceable. Most important, the viral HEV load and the resulting infectious dose is not determined. Taken into consideration only the individual cases included in Tables S1 and S2 where the viral load infused is available, 39 patients received blood products containing HEV, of whom 28 patients develop TT-HEV infection.

**Figure 3:**
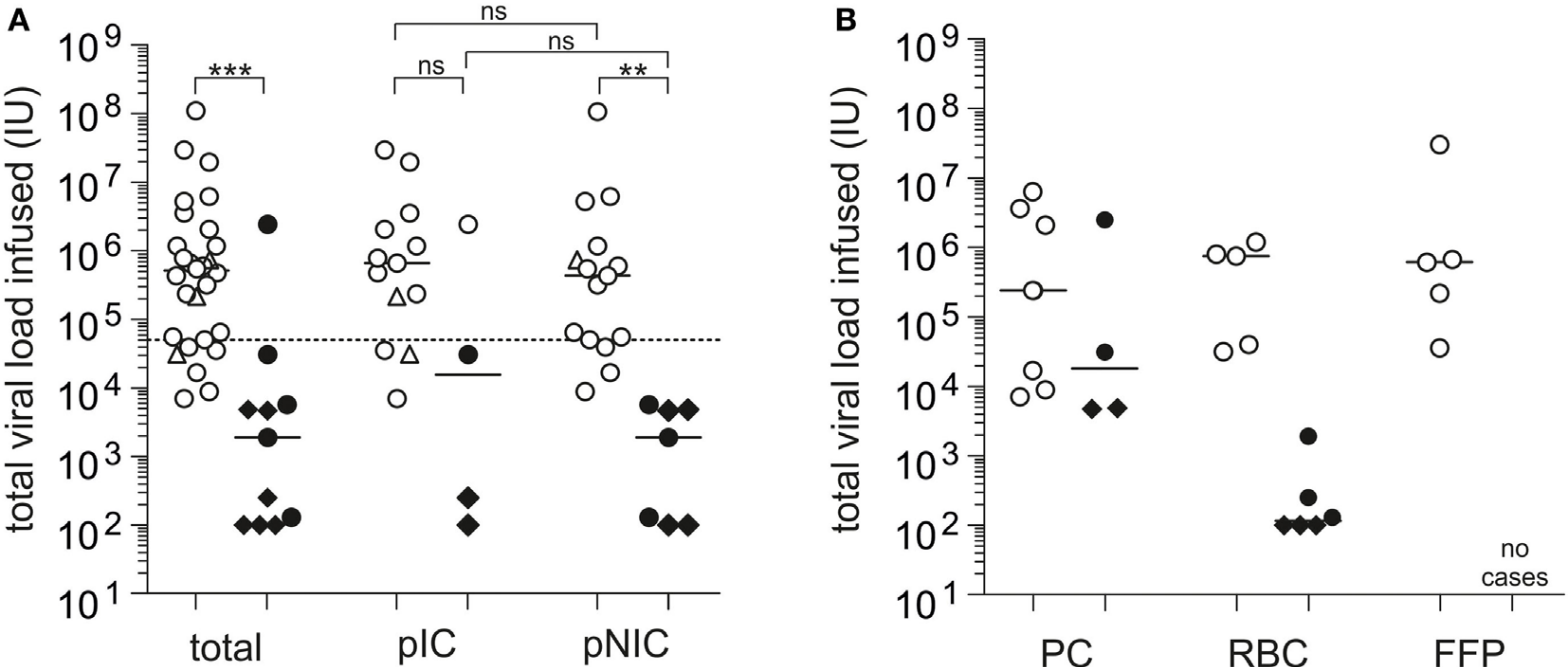
Systematic case review analysis of the total viral load transfused observed in individual case studies (Tables S1 and S2). **(A)** Displayed is the total viral load transfused resulting in posttransfusion hepatitis E virus (HEV) infection or no posttransfusion HEV infection, independently from and depending on the immune status of the recipients (*n* = 39). pIC, possibly immunocompromised; pNIC, possibly not immuno-compromised. **(B)** Displayed is the total viral load transfused resulting in posttransfusion HEV infection or no posttransfusion HEV infection depending on the transfused blood product (*n* = 25). RBC, red blood cell concentrates; PC, apheresis or pooled PCs; FFP, fresh frozen plasma. ◊: values specified with <IU/mL, viral loads for these cases are placed at the maximum possible value, ∆: estimated infectious dose, solid bars indicate median viral load. The solid horizontal line represents median values, and the dotted horizontal line represents the minimum infectious dose. White symbols: HEV infection and black symbols: no HEV infection. ***p < 0.0001, **p = 0.0002, and *p < 0.05, ns, not significant.

Tedder and colleagues performed an estimation of the infectious dose of the individual blood product types involved in the UK study in a subsequent analysis, demonstrating that components causing TT-HEV infection had a considerably higher median infectious dose of 1.44E+06 IU than components not causing TT-HEV infection [median total viral load transfused: 2.40E+04 IU (2, 24)]. Accordingly to this study, our systematic case review analysis showed a significant difference in the median viral load transfused between HEV-infected (median: 5.20E+05 IU, this study) and non-infected patients (median: 1.91E+03 IU, *p* < 0.0001, Figure 3A). Statistical significant differences in the median viral load transfused were also observed between HEV-infected (median: 4.40E+05 IU) and non-infected non-immunocompromised patients (median: 1.91E+03 IU, *p* = 0.0002), whereas no differences were observed between HEV-infected (median: 4.80E+05 IU) and non-infected immunocompromised patients (median: 1.55+04 IU, *p* = 0.1006). When the immune status of the recipient, which was mentioned to have a major impact on the actual risk of TT-HEV infection, was also taken into account, no differences were observed in the median viral load transfused between immunocompromised and non-immunocompromised patients, independently from the infection outcome (HEV-infected, *p* = 0.6286; non-HEV-infected, *p* = 0.5044). The lowest infectious dose resulting in TT-HEV infection observed in general was 7.05E+03 IU. When the type of blood product was considered, the lowest infectious dose transfused was 7.05E+03 IU for PCs without subdivision of APC and PPCs, 3.16E+04 IU for RBCs, and 3.60E+04 IU for FFPs (Figure 3B). Tedder et al. demonstrated that the lowest dose of virus resulting in an infection was 2.00E+04 IU, whereby only 55% of the components containing this dose transmitted an infection (2). Among non-transmitting components, 60% contained or exceeded this infectious dose. Satake et al. summarized that infusion of total viral loads between 2.00E+04 IU and 2.60E+05 IU can occur without HEV transmission (29). In our systematic case review analysis, all components with a viral load >5.00E+04 IU caused infection (Figure 3A), independently from the immune status of the recipient; however, only one of the non-transmitting components exceeded this value. Furthermore, the pretransfusion serostatus of recipients receiving HEV-contaminated blood products might have an impact on the development of HEV infection. With the exception of three cases in the study by Hewitt et al. (24) and two additional cases (29, 38, 39, 46), all described cases had a seronegative pretransfusion status. Future studies including IgG positive pretransfusion cases might contribute to the assessment of a protective effect of previous experienced HEV infection or the effectiveness of future available vaccination of donors and/or at risk recipients.

In addition to the cases mentioned in Table S1 in Supplementary Material, Arankalle et al. described two cases of putative, but in our opinion unlikely, posttransfusion hepatitis. HEV infection was assumed due to seroconversion of both patients within weeks after transfusion, but no HEV RNA was detected in patients for comparative sequence analysis. Additionally, donor screening of five of the six involved donations revealed no HEV RNA (6). In Japan, seven further posttransfusion hepatitis E cases (six cases of RBC transfusion, one case of PC transfusion) were detected, according to the official announcement of the Japanese Red Cross Society, but no information on either donor or patient antibody status or the infectious dose were available (26). The German authorities also announced four further posttransfusion hepatitis E cases (two RBC, two PC) in their actual hemovigilance report so far, without detailed case information (47).

The lack of a small animal model and efficient cell culture system has hampered the study of HEV replication, pathogenesis, and infectious dose determination. HEV isolates of genotypes 3 and 4 have been adapted to grow *in vitro*, but HEV cell culture is inefficient and limited, and requires genetic modifications of the HEV isolates (48–51). Experimental HEV infection in the rhesus monkey model led to acute hepatitis E after transfusion of 10 mL plasma from a HEV-infected donor (52). Immune-deficient human-liver chimeric mouse also serves as an appropriate model to study HEV genotype 1 and 3 infection, virus–host interactions, and drug efficacy (53, 54). For example, chronic HEV infection was observed after intrasplenic injection of HEV-GT1-containing preparation with an infectious dose of 2.5E+05 IU (53). These models could serve as a starting point to determine the infectivity and pathogenicity of HEV. However, it is currently questionable whether these models are faithful representation of human infection and could answer the question of infectious dose in humans.

The plasma proportion of the transfused blood product seemed to affect the risk for TT-HEV at most, but so far no information is available on the partitioning of HEV into the different components from a single blood donation (9). It is conceivable that manufacturing processes during the fractionation of whole blood might result in lower viral loads than what is expected on the basis of viral plasma load of blood donors and the assumed residual plasma content. We have shown that the fractionation process in our blood transfusion facility does not considerably reduce the concentration of viral RNA, but this may not generally valid for other production processes.

The question remains as to which screening strategy is necessary and practicable. Screening may constitute a universal approach to include all blood products or a selective screening can be performed for only the products that would be used in at-risk patients. This issue is primarily influenced by two sides: the hospital-sided clear definition of at-risk patients and the logistic implications for the order of blood products, and supply and availability, managed by the blood establishment. The second question is whether minipool screening of up to 96 samples or ID testing is necessary. We would submit that the required detection limit, which need to be derived from the infectious dose, plays an important role for the second issue. Thus, the decision for ID or pool NAT depends on logistic and costs, which are in part dictated by the required sensitivity. Table 2 summarizes the currently available commercially HEV NAT-screening assays including the analytical sensitivities. The analytical sensitivity (95% LOD) of HEV NAT assays ranges from 4.7 to 18.6 IU/mL, and all assays used for blood screening detected positive donations of all genotypes 1 to 4 and demonstrated a good performance in routine testing (Table 2). In most settings, the Procleix HEV (Grifols) is used in individual testing (ID NAT), where the 95% LOD was determined to be 5.5–12.78 IU/mL, which is slightly different than that of the manufacturer’s value (15, 55). The disadvantage of current commercial HEV NAT assays is their requirement of special screening platforms that are fully integrated and automated, and not as flexible as open NAT platforms. For this reason, we introduced routine minipool HEV NAT screening (96 donations) using an in-house testing regime in our transfusion facility in January 2015. The setting of HEV NAT using RealStar HEV RT-PCR Kit 1.0 [Altona Diagnostics, 95% LOD 4.7 IU/mL (CI: 3.6–7.6, 452 IU/mL) per single donation] is compatible to the virus NAT screening used in our blood transfusion service (13, 56). Our HEV NAT is comparable with commercial HEV NAT-screening methods (Table 2) in spite of a lower level of automation and throughput. The novel automation platform AltoStar allows ID NAT testing or alternatively a higher automation grade for pool NAT. It is to be noted that the sensitivity of the RealStar HEV RT-PCR Kit strongly depends on the nucleic-acid extraction method used, ranging from 4.7 to 37.8 IU/mL (13, 55, 56). In our screening setting, we use the fully automated nucleic-acid extraction method Chemagic Viral DNA/RNA Kit that allows the processing of large plasma volumes (4.8 mL). Compared with the other commercial HEV NAT-screening methods from GFE Blut, Roche, and Grifols, the processed sample volume of our method is 3.7-fold, 5.6-fold, and 9.1-fold higher, respectively, resulting in a considerably higher number of HEV plasma-equivalents per PCR reaction. Therefore, this combination is, to the best of our knowledge, the most sensitive HEV NAT. It does not fully meet the sensitivity of 100 IU HEV RNA/mL recommended by the German authorities, but at present, it is not clear whether this sensitivity is absolutely necessary. Our minipool screening strategy aims to identify high viremic donors and will cover at least a large part of viremic phases (22, 57). The European medicine agency so far has also recommended minipool screening in their reflection paper on hepatitis E (9). However, it remains to be seen in the future whether all relevant viremic phases that could result in TT-HEV infections will be detected.

**Table 2:** Overview of currently commercially available hepatitis E virus (HEV) NAT-screening methods.

Kit name	RealStar HEV RT-PCR Kit 1.0	Cobas HEV test	Procleix HEV assay	HEV NAT kit

Manufacturer	Altona Diagnostics	Roche Diagnostics	Grifols	GFE Blut
Automation	No	Full automation cobas^®^ 6800/8800 Systems	Full automation Procleix^®^ Panther^®^ System	Full automation PoET System
FDA/CE-IVD	No/yes	No/yes	No/yes	No/yes

**Sample preparation/nucleic-acid extraction**				
Virus enrichment pre-extraction	No	No	No	No
Maximal MP size	96	Depending on regional regulation	12	Up to 96
Nucleic-acid extraction procedure	Chemagic Viral RNA/DNA Kit on MSM-I (4.8-mL protocol)	Magnetic glass particles for fully automated NA-extraction	Target-specific extraction––magnetic microparticles capture viral nucleic acids with viral-specific capture oligonucleotides	Fully automated magnetic bead extraction
Processed sample volume (plasma mL)	4.8	0.85	0.525	1.3
Elution volume (μL)	100	50	n.a., single-tube format	90
Plasma-equivalents (mL)/PCR (%)	1.2 (100)	0.425 (35)	0.525 (44)	0.433 (36)

**NAT/detection**				
Principle of NAT detection	RT-PCR, TaqMan probes	RT-PCR, TaqMan probes	Transcription-mediated amplification	RT-PCR, TaqMan probes
NAT instrument	Rotorgene Q	cobas^®^ 6800/8800 Systems	Procleix^®^ Panther^®^ System	PoET System
Target (gene region)	ORF3	5′UTR	n.a.	n.a.
Eluat/PCR volume (μL/μL)	25/50	25/50	100% of the sample is processed and used in the amplification reaction	30/75 or 10/25

**Test specifications**				
Analytical sensitivity (95% LOD IU/mL)	4.7 (451.2–96 pool)	18.6	7,88	8.2 (787.2–96 pool, 75-µL PCR)
Specificity	100% Genotype 1–4	100% Genotype 1–4	99,98% Genotype 1–4	100% Genotype 1–4

**Accomplishment**				
Hands on time	30 min	15 min	15 min	n.a.
Time to result	4 h	3 h	3.5 h	5 h
Throughput	960 results (10 pools of 96 samples) in 4 h	96 results (94 pools plus 2 controls) in 3 h 384 results (376 pools plus 8 controls) in 8 h shift (cobas^®^ 6800 System)	5,775 results (275 pools of 16 samples) in 8 h 10,500 results (500 pools of 16 samples) in 12 h	Depending on configuration. e.g., 8,448 in 5 h, 16,896 in 9 h (176 pools of 96 samples)
Remarks	Automation on AltoStar system for ID NAT pending		Intended use includes cadaveric (non-heart beating) donors	Preliminary data; IVD certification pending.

## Author Contributions

JD and TV designed the study, analyzed and interpreted the data, and drafted the manuscript. CK designed the study and revised the manuscript critically. All authors contributed to drafting the text and approved the manuscript.

## Conflict of Interest Statement

The author declares that the research was conducted in the absence of any commercial or financial relationships that could be construed as a potential conflict of interest.
