# Supplementary material for: Transfusion-Transmitted Hepatitis E: NAT Screening of Blood Donations and Infectious Dose
Source: Front Med (Lausanne). 2018 Feb 1;5:5. doi: 10.3389/fmed.2018.00005 (PMC5799287; doi:10.3389/fmed.2018.00005)
Supplement: Supplementary file 1 [file Table_1.PDF]

SUPPLEMENTAL MATERIAL

Table S1: Cases of proven and probable transfusion-transmitted HEV infection: review of the literature (2004-2017)

RECIPIENT					DONOR				
**age (y), sex**	**disease/therapy[1]**	**IgM/IgG pre-/post transfusion[2]**	**outcome after transfusion, (follow-up period)**	**involved blood product[3]**	**age (y), sex**	**viral load (VL) genotype (GT) infectious dose (ID)**	**IgM/ IgG**	**Country, year**	**Ref.**
25, F	mitral valve replacement (pNIC)	pre: N/N<br>post: P/P[4]	HEV infection, no symptoms (3 mo.)	NS	NS	VL/GT/ID: NS	P/N	India 1994-95	(25)
35, F	splenectomy for hemolytica anemia (pNIC)	pre: N/N<br>post: P/P[4]	HEV infection, no symptoms (3 mo.)	RBC	NS	VL/GT/ID: NS	P/N	India 1994-95	(25, 26)
45, M	GI bleeding (pNIC)	pre: N/N<br>post: N/P[4]	icteric HEV infection, (3 mo.)	RBC	NS	VL/GT/ID: NS	N/N	India 1994-95	(25, 26)
31, M	hemodialysis (pNIC)	pre: N/N<br>post: N/P	acute HEV infection (21 d)	RBC	NS	VL/ID: NS<br>GT: 3	N/N	Japan 1979	(27)
21, M	T-cell lymphoma, bone marrow transplantation (IC)	pre: (N/N)<br>post: N/N (limited immune response)	HEV infection (167 d)	RBC	NS	VL: NS<br>GT: 3<br>ID: 1.2E+06 IU	NS	Japan 1999	(28, 29)
2 patients NS	thrombotic thrombocytopenic purpura (pNIC)	pre: N/N<br>post: P/P[4]	acute HEV infection (6 mo.)	SDP	NS	VL/ID: NS<br>GT:3a<br>pool of < 25,000 single-donor units	NS	Canada 2001-2003	(30)
67, M	valve replacement surgery (NIC)	pre: N/N<br>post: P/P	acute HEV infection (134 d)	FFP	24, F	VL: NS<br>GT: 4<br>ID: 6.1E+05 IU	N/N	Japan 2002	(29, 31)
64, M	non-Hodgkin's lymphoma (IC)	pre: N/N<br>post: N/P	acute HEV infection (130 d)	PC	39, M	VL:1.26 E+03 cop/ml<br>GT: 4<br>ID: 2.4E+05 IU	N/N	Japan 2004	(29, 32)
70's, M	re-thoracotomy for hemostasis after myocardial infarction (pNIC)	pre: N/N<br>post: P/P	acute HEV infection, clearance without therapy (120 d)	PC	NS	VL: NS<br>GT: 3<br>ID: 6.3E+06 cop	NS	Japan 2005	(33)
72, M	angina (NIC)	pre: (N/N)<br>post: P/P	acute HEV infection (NS)	NS	NS	VL: NS<br>GT: 3<br>ID:5.3E+06 IU	NS	Japan 2005	(29)

RECIPIENT					DONOR				
**age (y), sex**	**disease/therapy[1]**	**IgM/IgG pre-/post transfusion[2]**	**outcome after transfusion, (follow-up period)**	**involved blood product[3]**	**age (y), sex**	**viral load (VL) genotype (GT) infectious dose (ID)**	**IgM/ IgG**	**Country, year**	**Ref.**
58, M	angina (NIC)	pre: (N/N) post: P/P	acute HEV infection (NS)	NS	NS	VL: NS GT: 3 ID: 1.2E+06 IU	NS	Japan 2005	(29)
7, M	kidney, rhabdoid tumor (pIC)	pre: N/N post: P/N	acute HEV infection (~84 d)	RBC	24, M	VL/ID: NS GT: 3f	N/N	France 2005	(34)
65, M	testicle B-cell lymphoma (pIC)	pre: N/N post: P/N[4]	acute HEV infection (~130 d)	RBC	in 40's, M	VL/ID: NS GT: 3	P/P	UK, NS	(35)
74, M	epidural abcess, ARDS, sepsis, acute renal failure (NIC)	pre: (N/N) post: N/P	acute HEV infection (NS)	NS	NS	VL: NS GT: 3 ID: 3.2E+05 IU	NS	Japan 2008	(29)
82, M	prostate cancer (NIC)	pre: (N/N) post: P/P (RNA N)[4]	probable HEV infection (NS)	NS	NS	VL: NS GT: 3 ID:1.1E+08 IU	NS	Japan 2008	(29)
66, M	gastric cancer, dialysis (NIC)	pre: (N/N) post: N/P	acute HEV infection (NS)	NS	NS	VL: NS GT:3 ID: 5.6E+05 IU	NS	Japan 2008	(29)
81, M	ischemic heart disease, chronic autoimmune thrombocytopenia/anemia (corticoid therapy, pIC)	pre: N/N post: P/P	acute HEV infection, clearance after withdrawn of corticosteroid therapy (~7 mo.)	PPC	53, F	VL: 1.7E+03 IU/ml GT: 3f ID: 1.7E+04 IU[5]	N/N	France 2011	(23)
27, F	acute promyelocytic leukemia, severe DIC and sepsis (IC)	pre: (N/N) post: N/P	late onset HEV infection (4 months post transfusion)	FFP	NS	VL: NS GT: 3 ID: 3.6E+04 IU	NS	Japan, 2011	(26, 29)
70, M	myelodysplastic syndrome, lung abcess, pneumonia (NIC)	pre: N/N post: P/P	acute HEV infection (50 days)	RBC	NS	VL: 1.2 E+03 cop/ml GT: 3 ID: 4.0E+04 IU	N/N	Japan 2011	(29, 36)
55, F	myelofibrosis, immune thrombocytopenic purpura (NIC)	pre: (N/N) post: P/P	acute HEV infection (NS)	NS	NS	VL: NS GT: 3 ID: 6.6E+04 IU	NS	Japan 2011	(29)
77, M	abdominal trauma (NIC)	pre: (N/N) post: ID/P (RNA N)[4]	probable HEV infection (NS)	NS	NS	VL: NS GT: 3 ID:5.1E+04 IU	NS	Japan 2011	(29)

RECIPIENT					DONOR				
**age (y), sex**	**disease/therapy[1]**	**IgM/IgG pre-/post transfusion[2]**	**outcome after transfusion, (follow-up period)**	**involved blood product[3]**	**age (y), sex**	**viral load (VL) genotype (GT) infectious dose (ID)**	**IgM/ IgG**	**Country, year**	**Ref.**
37, F	burkitt lymphoma (IC)	pre: (N/N)<br>post: ID/ID	chronic HEV infection despite ribavirin therapy (8 mo.)	PC	NS	VL: NS<br>GT: 3<br>ID: 2.1E+06 IU	NS	Japan 2011	(29, 37)
61, F	primary bilary cirrhosis, liver transplantation (IC)	pre: N/WP<br>post: P/P	chronic HEV infection (1 y)	FFP	NS	VL: NS<br>GT: 3<br>ID: 6.7E+05 IU	NS	Japan, 2012	(29, 38, 39)
72, M	diabetes mellitus nephropathy, bypass surgery (NIC)	pre: NS<br>post: ID/ID	acute HEV infection (NS)	NS	NS	VL/GT: NS<br>ID:4.4E+05 IU	NS	Japan 2012	(29)
81, F	myelodysplastic syndrome (NIC)	pre: (N/N)<br>post: P/P	acute HEV infection (NS)	NS	NS	VL: NS<br>GT: 3<br>ID:5.6E+04 IU	NS	Japan 2012	(29)
36, M	chronic renal failure, kidney transplantation (pIC)	pre: (N/N)<br>post: WP/N	chronic HEV infection, ribavirin therapy (>1 y)	ICT plasma	32, F	VL/ID: NS<br>GT: 3f	NS	France 2012	(40)
61, M	alcoholic liver cirrhosis, liver transplantation (pIC)	pre: N/N<br>post: N/N (time of infection)	acute or chronic HEV infection, ribavirin therapy (~1 y)	ICT plasma					
55, M	HBV-induced liver cirrhosis, liver transplantation (pIC)	pre: (N/N)<br>post: N/N	acute HEV infection, clearance ribavirin therapy (12 mo.)	RBC	41, M	VL: 3.16E+03 IU/ml<br>GT: 3c<br>ID: 3.16 E+04 IU[5]	N/N	France 2012	(41)
47, M	immuno-compromised (IC)	pre: NS<br>post: P/P	chronic HEV infection (6 mo.)	APC	In 40´s, M	VL: 120 IU/ml<br>GT: 3f<br>ID: 7.06IU – 8.89 E+03 IU	N/N	Germany 2013	(42)
6, M	not immuno-compromised (NIC)	pre: NS<br>post: WP/P[4]	probable HEV infection (8 mo.)						
41, M	liver cirrhosis, liver transplantation (IC)	pre: N/N<br>post: P/P	chronic HEV infection (> 6 mo.)	PC	NS	VL: 1.8E+04 cop/ml<br>GT: 3b<br>ID: 3.6E+06 IU	NS	Japan, 2014	(29, 38, 43)
6, M	liver transplantation (IC)	pre: NS<br>post: NS	acute HEV infection, clearance ribavirin therapy (5 mo.)	FFP	NS	VL: 947 IU/ml<br>GT: 3chi group<br>ID: 2.2E+05 IU[5]	NS	Australia 2014	(44)

<table>
<tr><th colspan="5">RECIPIENT</th><th colspan="3">DONOR</th><th colspan="2"></th></tr>
<tr><th>age (y), sex</th><th>disease/therapy[1]</th><th>IgM/IgG pre-/post transfusion[2]</th><th>outcome after transfusion, (follow-up period)</th><th>involved blood product[3]</th><th>age (y), sex</th><th>viral load (VL) genotype (GT) infectious dose (ID)</th><th>IgM/ IgG</th><th>Country, year</th><th>Ref.</th></tr>
<tr><td>61, M</td><td>axillofemoral bypass (pNIC)</td><td>pre: (N/N)
post: P/P</td><td>acute HEV infection, clearance without therapy (3 mo.)</td><td>RBC</td><td>41, M</td><td>VL: 7.5E+04 IU/ml
GT: 3f
ID: 7.5E+05 IU[5]</td><td>N/N</td><td>Spain 2015</td><td>(45)</td></tr>
<tr><td>67, M</td><td>hodgkin's lymphoma (IC)</td><td>pre: (N/N)
post: P/P</td><td>acute HEV infection (NS)</td><td>NS</td><td>NS</td><td>VL: NS
GT: 3
ID: 2.0E+07 IU</td><td>NS</td><td>Japan 2015</td><td>(29)</td></tr>
<tr><td>52, F</td><td>multiple myeloma, stem cell transplantation (IC)</td><td>pre: (N/N)
post: P/P</td><td>acute HEV infection (NS)</td><td>NS</td><td>NS</td><td>VL: NS
GT: 3
ID: 4.8E+05 IU</td><td>NS</td><td>Japan 2015</td><td>(29)</td></tr>
<tr><td>33, M</td><td>stem cell transplant recipient (IC)</td><td>pre: NS
post: NS[4]</td><td>chronic HEV infection (>1 y)</td><td>RBC</td><td rowspan="2">NS</td><td>VL: 10E+05 IU/ml
GT: 3
ID: 8.0E+05 IU</td><td rowspan="2">N/N</td><td rowspan="2">Germany 2016</td><td rowspan="2">(46)</td></tr>
<tr><td>71, M</td><td>heart transplantation (IC)</td><td>pre: NS/P
post: NS/P[4]</td><td>chronic HEV infection (> 3 mo.)</td><td>FFP</td><td>VL: 10E+05 IU/ml
GT: 3
ID: 3.0E+07 IU</td></tr>
</table>

NS: not specified,
[1] IC: immunocompromised, NIC: not immunocompromised (classification by author); pIC/pNIC probable (not) immunocompromised (classification by the authors of this study)
[2] N: negative, P: positive, WP: weak positive, (N/N): assumed pre-transfusion status derived from post-transfusion status
[3] SDP: solvent-detergent-treated plasma, PC: platelet concentrate, APC: apheresis-derived platelet concentrate, RBC: red blood cell concentrate, FFP: fresh frozen plasma, ICT plasma: plasma treated by intercept pathogen reduction technologies
[4] no HEV sequence comparison of donor and recipient strain
[5] estimated infectious dose, RBC: assumption of a residual plasma volume of 10 ml plasma per RBC (2, 11), FFP: assumption of a mean FFP volume of 287 ml containing 230 ml human plasma (this study), PPC: assumption of a residual plasma volume of 10 ml plasma per PPC (11)
