# Supplementary material for: Transfusion-Transmitted Hepatitis E: NAT Screening of Blood Donations and Infectious Dose
Source: Front Med (Lausanne). 2018 Feb 1;5:5. doi: 10.3389/fmed.2018.00005 (PMC5799287; doi:10.3389/fmed.2018.00005)
Supplement: Supplementary file 2 [file Table_2.PDF]

SUPPLEMENTAL MATERIAL

Table S2: Cases of transfusion of HEV containing blood products not resulting in transmitted HEV infection: review of the literature and data from this study

RECIPIENT					DONOR				
**age (y), sex**	**disease/therapy[1]**	**IgM/IgG pre-/post transfusion[2]**	**outcome after transfusion, (follow-up period)**	**involved blood product[3]**	**age (y), sex**	**viral load (VL) genotype (GT) total viral load infused (TVLI)**	**IgM/ IgG**	**Country, year**	**Reference**
55, F	primary biliary cirrhosis and choledocholithiasis (pNIC)	pre: N/N<br>post: N/N	no HEV infection	PC[4]	in 40's, M	VL/TVLI: NS<br>GT: 3	P/P	UK, NS	(35)
N.N.	lymphoma (pIC)	pre: N/N<br>post: N/N	no HEV infection (130 d)	RBC	24, F	VL/TVLI: NS<br>GT: 4	N/N	Japan 2002	(29, 31)
40's, F	gynecological surgery (pNIC)	pre: NS/P<br>post: N/P	no HEV infection (NS)	RBC	NS	VL/TVLI: NS<br>GT: 3	NS	Japan, 2011	(26)
71, M	immuno-compromised (IC)	pre: NS<br>post: NS/N	no HEV infection (5 months)	APC	In 40´s, M	VL: 495 IU/ml<br>GT: 3f<br>TVLI: 3.09 E+04 IU	N/N	Germany 2013	(42)
54, M	alcoholic, melaena (pNIC)	pre: NS<br>post: N/N	no HEV infection (58 d)	RBC	32, M	VL: < 10 IU/ml<br>GT: NS<br>TVLI: <100 IU	N/N	Denmark 2015	(14)
62, M	acute myeloic leukemia (pIC)	pre: NS<br>post: N/N	no HEV infection (30, 210 d)	RBC	27, M	VL: <10 IU/ml<br>GT: NS<br>TVLI: <100 IU	P/P	Denmark 2015	
87, M	orthopedic surgery (pNIC)	pre: NS<br>post: N/P	no HEV infection (46 d)	RBC	48, M	VL: 191 IU/ml<br>GT: NS<br>TVLI: 1.91E+03 IU/ 5.73E+03 IU	P/P	Denmark 2015	
0, F	cardiac surgery (pNIC)	pre: NS<br>post: N/N	no HEV infection (36 d)	Cryo				Denmark 2015	
35, M	alcoholic cirrhosis (pNIC)	pre: NS<br>post: N/N	no HEV infection (60 d)	RBC	53, M	VL: 13 IU/ml<br>GT: 3<br>TVLI: 3.51E+03 IU	N/N	Denmark 2015	
74, F	ischemic heart disease, chronic kidney disease (pNIC)	pre: NS<br>post: N/N	no HEV infection (124 d)	RBC	40, M	VL/GT/TVLI: NS	N/N	Denmark 2015	
71, M	hemodialysis treatment with intravenous IgG (pNIC)	pre: NS<br>post: N/N	no HEV infection (112 d)	RBC	48, F	VL: <10 IU/ml<br>GT: NS<br>TVLI: <100 IU	P/P	Denmark 2015	

RECIPIENT					DONOR				
**age (y), sex**	**disease/therapy[1]**	**IgM/IgG pre-/post transfusion[2]**	**outcome after transfusion, (follow-up period)**	**involved blood product[3]**	**age (y), sex**	**viral load (VL) genotype (GT) total viral load infused (TVLI)**	**IgM/ IgG**	**Country, year**	**Reference**
23, M	heart transplantation (pIC)	pre: N/N<br>post: N/N	no HEV infection (134 d)	RBC	23, M	VL: <25 IU/ml<br>GT: 3<br>TVLI: <250 IU	N/N	Germany 2015	this study
76, M	heart valve failure, atrial fibrillation (pNIC)	pre: N/N<br>post: N/N	no HEV infection (35 d)	APC1	24, F	VL: <25 IU/ml<br>GT: 3<br>TVLI: <4.68E+03 IU	N/N	Germany 2016	
54, M	left ventricular heart failure (pNIC)	pre: N/N<br>post: N/N	no HEV infection (50 d)	APC2		VL: <25 IU/ml<br>GT: 3<br>TVLI: <4.86E+03 IU	N/N	Germany 2016	
26, F	hypertrophic cardiomyopathy (pNIC)	pre: N/N<br>post: N/N	no HEV infection (16 d)	2 APC	55, M	VL: 27.8 IU/ml<br>GT: 3<br>TVLI: 1.08E+04 IU	N/N	Germany 2016	
72, M	arrhythmia (pNIC)	pre: N/N<br>post: NA	no HEV infection (NA)	2 APC	55, M	VL: 69.4 IU/ml<br>GT: 3<br>TVLI: 2.71E+04 IU	N/N	Germany 2016	
79, F	leukemia (pIC)	pre: N/N<br>post: NA	no HEV infection (NA)	2 APC	29, M	VL: <25 IU/ml<br>GT: 3<br>TVLI: 7.97E+03 IU	N/N	Germany 2017	
61, F	acute myeloid leukemia (pIC)	pre: NS/P<br>post: NS/P	no HEV infection (49 d)	PPC	NS	VL: 10E+05 IU/ml<br>GT: 3<br>TVLI: 2.5E+06 IU	N/N	Germany 2016	(46)

NS: not specified, N: negative, P: positive, NA: not applicable

[1] IC: immunocompromised, NIC: not immonocompromised (classification by author); pIC/pNIC probable (not) immunocompromised (classification by the authors of this study)

[2] serostatus IgG: seroconversion: pre-transfusion IgG negative, post-transfusion IgG positive, positive: pre-transfusion IgG positive, post-transfusion IgG positive, indeterminate: no clear indication

[3] PC: platelet concentrate, APC: apheresis-derived platelet concentrate, RBC: red blood cell concentrate, Cryo: cryoprecipitate pool

[4] platelets not suspended in HEV donor's plasma, no HEV neutralizing antibodies
